# Immunotherapy in the Treatment of Metastatic Melanoma: Current Knowledge and Future Directions

**DOI:** 10.1155/2020/9235638

**Published:** 2020-06-28

**Authors:** Massimo Ralli, Andrea Botticelli, Irene Claudia Visconti, Diletta Angeletti, Marco Fiore, Paolo Marchetti, Alessandro Lambiase, Marco de Vincentiis, Antonio Greco

**Affiliations:** ^1^Department of Sense Organs, Sapienza University of Rome, Viale del Policlinico 155-00161 Rome, Italy; ^2^Department of Clinical and Molecular Medicine, Sapienza University of Rome, Viale del Policlinico 155-00161 Rome, Italy; ^3^Institute of Cell Biology and Neurobiology, IBCN-CNR, Rome, Italy; ^4^Department of Oral and Maxillofacial Sciences, Sapienza University of Rome, Viale del Policlinico 155-00161 Rome, Italy

## Abstract

Melanoma is one of the most immunologic malignancies based on its higher prevalence in immune-compromised patients, the evidence of brisk lymphocytic infiltrates in both primary tumors and metastases, the documented recognition of melanoma antigens by tumor-infiltrating T lymphocytes and, most important, evidence that melanoma responds to immunotherapy. The use of immunotherapy in the treatment of metastatic melanoma is a relatively late discovery for this malignancy. Recent studies have shown a significantly higher success rate with combination of immunotherapy and chemotherapy, radiotherapy, or targeted molecular therapy. Immunotherapy is associated to a panel of dysimmune toxicities called immune-related adverse events that can affect one or more organs and may limit its use. Future directions in the treatment of metastatic melanoma include immunotherapy with anti-PD1 antibodies or targeted therapy with BRAF and MEK inhibitors.

## 1. Introduction

Melanoma is an immunologic malignancy characterized by higher prevalence in immune-compromised patients, evidence of brisk lymphocytic infiltrates in both primary tumors and metastases, documented recognition of melanoma antigens by tumor-infiltrating T lymphocytes and, most important, evidence that melanoma responds to immunotherapy [1–3].

Immunotherapy is one of the most efficient therapeutic strategies in melanoma because of the high immunogenicity of this tumor. The mechanisms of action of immunotherapy are focused on specific targets of the counter-regulatory mechanisms of the immune response [4–8]. However, immunotherapy is also associated with immune-related adverse events(irAEs) that represent tissue-specific dysimmune inflammatory responses [9–14].

This review paper discusses current knowledge and future directions in melanoma immunogenicity and immunotherapy.

## 2. Metastatic Melanoma

The incidence of cutaneous melanoma has rapidly increased in the past decades. Melanoma is the ninth most common malignancy and the second for mortality. Every year, there are nearly 100,000 new cases of melanoma in the United States, and about 9,000 patients die of this cancer [15]. Despite prevention campaigns, melanoma incidence has increased at a faster rate compared to most other cancers, especially in young Caucasian women [16].

Melanoma patients with distant metastases show a 5-year survival rate of 23%, making metastasis the leading cause of melanoma-associated deaths [17].

Several factors are involved in the pathogenesis of melanoma, including environmental, genetic, and immunological ones [18–20]. Of these, research has mainly focused on the activation of the immune system, especially for the possibility of developing specific targeted therapies [1, 2, 18, 19].

### 2.1. Environmental Factors

**Figure 1:**
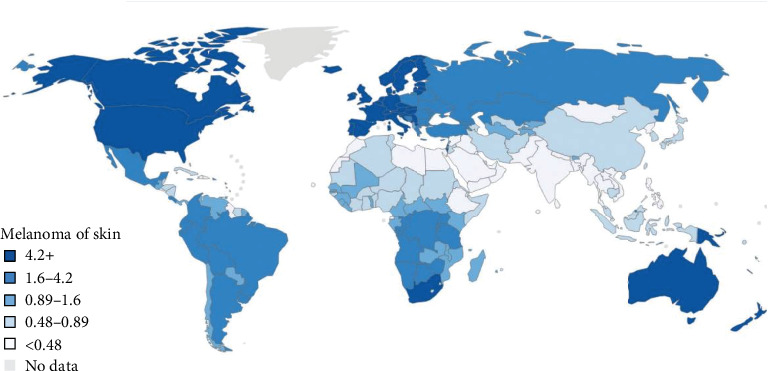
Global incidence of Melanoma of skin. From Matthews NH et al. “Epidemiology of Melanoma” Cutaneous Melanoma: Etiology and Therapy. 2017 [26].

Studies have revealed that many factors may favor the development of melanoma; among them, the environment and the exposure to ultraviolet (UV) rays play an important role [21–24]. The incidence of melanoma varies by geographic location among people of the same ethnicity. Different locations can translate into differences in atmospheric absorption, latitude, altitude, cloud cover, and seasonality, thus influencing incident UV radiation [1, 2]. In 1956, Lancaster found increasing melanoma mortality rates with increasing proximity to the equator, a phenomenon he termed the “latitude gradient” [25]. Since then, similar trends of melanoma incidence have been reported around the world. In the lowest latitudes, melanoma annual incidence tends to be higher than in higher latitudes [26] (Figure 1).

Differences in altitude have also been suggested to have a role in melanoma incidence. In countries with both high- and low-latitude locations, higher altitudes have been associated with higher melanoma incidence. In fact, the UV irradiance is associated with higher altitude; furthermore, with higher altitude, there are also changes in ozone absorption, decreased cloud cover, and increased surface reflectance from snow cover which can also increase UV radiation [27].

### 2.2. Genetic Factors

Genetics factors may have a role in the pathogenesis of melanoma. In 2005, Uhara et al. reported an elevate detection of the BRAF mutation in patients with melanoma without chronic sun-induced damage [28, 29]. Further research showed that nearly 40-50% of cutaneous melanomas have mutations in BRAF, a gene that belongs to the family of mitogen activated protein kinase (MAPK) and codes for a serine/threonine protein kinase constituting part of RAS-RAF-MEK [30, 31]. BRAF activation induces the phosphorylation of extracellular signal-regulated kinases (ERK) that constitute the most common mutated isoforms in cancer [32]. The most common mutation is the V600E; in some cases, another mutation of BRAF named V600K has been described [33]. Some other gene mutations have been described in studies such as NRAS and KIT. Therefore, studies have revealed that there is a high mutation rate in melanoma when comparing to other common tumors [34, 35].

The recently identified high-risk variants such as BRCA1 Associated Protein 1 (BAP1), Protection of Telomeres Protein 1 (POT1), Adrenocortical Dysplasia (ACD), Telomeric Repeat-binding Factor-2 Interacting Protein (TERF2IP), and Telomerase Reverse Transcriptase (TERT) contribute to about 2% of melanoma's missing heritability [36].

Pastorino et al. also studied Ataxia-Telangiectasia Mutated (ATM) gene to define its role as a susceptibility gene for cutaneous melanoma. The authors reported a high percentage of deleterious ATM variants in melanoma families (3.3%) [37].

Recently, Casula et al. used a panel containing the same genes (with the exception of MITF) in a sample of Italian patients with cutaneous malignant melanoma and found a lower pathogenic variant rate based on the American College of Medical Genetics on Genomics variant classification (3%); in addition, a low level of heterogeneity in driver somatic mutations in subjects with numerous melanomas was reported [38].

### 2.3. Immunological Factors

**Figure 2:**
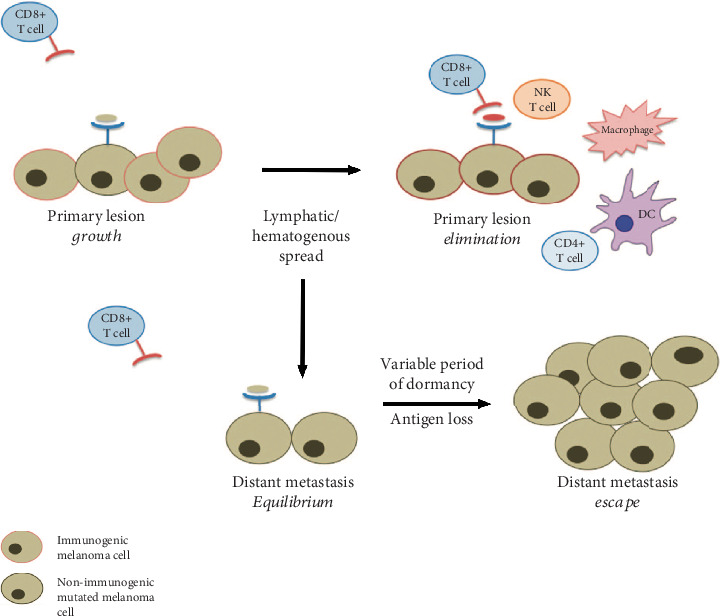
Suggested mechanisms of immunoediting in melanoma of unknown primary. From Gyorki et al., The delicate balance of melanoma immunotherapy. Clinical & Translational Immunology 2013 [34].

The high immunogenicity of melanoma is the base of the relationship between this cancer and the immune system [2, 5, 15, 18–20, 39]. The main characteristic of the immune system is to recognize the antigen as self or nonself. It is clear that the progression of melanoma is based on a lack of activation of the immune system and the ability of the tumor of doing the so-called “immune escape”. This is further supported by the evidence that some melanoma patients present with metastatic disease without an evident primary lesion; these cases are also known as “melanoma of unknown primary” and are based on immunoediting mechanisms [34, 40] (Figure 2).

The characteristics of an efficient immune system include a fast, nonspecific phase which activates the innate response and a second specific adaptive response [15, 41]. The response starts with the release of tumor antigens presented by antigen-presenting cells (APC) to T cells in the lymph nodes. APC are primarily dendritic cells (DC). Subsequently, T cells including CD8+ cytotoxic lymphocytes (CTL) reach the tumor where they recognize and kill malignant cells and contribute realizing more cancer antigens [32, 42]. Tumor-associated antigens, which are recognized by autologous antibodies and T cells, have been identified and classified in melanoma [16]. They can be characterized as differentiation antigens such as glycoprotein 100 (gp100), tyrosinase, and Melan-A.

A crucial role in the activation of the immune system is played by the costimulatory molecules. These molecules work to activate T cell response, amplify signals, or counteract T cell complex signals [43]. They represent one of the targets of immunotherapy in the treatment of metastatic melanoma [43].

The immune system is able to control the disease only in the initial phases, when the tumor is still in an early phase and defence mechanisms are still efficient; furthermore, the cancer causes the exhaustion of the immune system through a continuous antigenic stimulation. The exhaustion of the immune system and the immune escape allow melanoma to grow and become metastatic [15, 16, 18, 19, 39]. Briefly, melanoma cells can evade immune detection through a reduction of the expression of immunogenic tumor antigens, a reduction of the histocompatibility complex class I (MHC I), the alteration of the antigen process, the recruitment of the immunosuppressive cells such as T reg and suppressor cells derived from myeloid cells, and the reduction of immunosuppressive molecules such as TGFß, Vascular-Endothelial Growth Factor (VEGF), adenosine, or Indoleamin 2,3-dioxygenase enzyme (IDO) [32, 44–46].

## 3. Immunotherapy for Metastatic Melanoma

Immunotherapy is now considered a promising new approach for the treatment of metastatic melanoma [47–49], even if its role is a relatively late discovery for this malignancy. One of the main characteristics of immunotherapy is the resistance to radiation therapy and cytotoxic chemotherapy. In the past, the key drug for melanoma was Dacarbazine, with an overall response rate (ORR) of 10-20%; however, there were no differences between Dacarbazine monotherapy and a combined chemotherapy. No better results have been reported for radiotherapy. Despite the poor clinical results, these approaches have been the main drivers in melanoma treatment for decades [50–52].

The new immunotherapy is the treatment that has been most extensively studied in metastatic melanoma [53]. Immunotherapy can be divided into four main groups [17]. The first include biological medications such as cytokines, interferons, and granulocyte-monocyte colony-stimulating factors [54]. The second is the vaccination strategy based on peptide, on the whole protein, on virus, on DNA, or on DC [55]. The third group is based on adoptive cell therapy, which consists in the use of the so-called lymphokine-activated killer (LAK) cells, tumor-infiltrating lymphocytes (TIL), and other specific lymphocytes [56, 57]. The fourth group consists of immune checkpoint inhibitors; in the last few years, the immunologic origin of this malignancy has led to the discovery of antibodies directed to specific targets such as antiprogrammed cell death 1 (PD-1) and anticytotoxic T-lymphocyte-associated protein 4 (CTLA-4) [4, 5, 58]. These blockers have drastically increased and elongated the overall survival (OS) of metastatic melanoma [28].

### 3.1. Biological Immunotherapy

The biological immunotherapy was the first used in the treatment of metastatic melanoma to replace or complete the action of chemotherapy. The most common medications used in biological immunotherapy are high doses of interleukin 2 (IL-2) and interferons [59–61].

Biological immunotherapy is often used in combination with stereotactic radiotherapy [53, 62], vaccines or anti-CTLA-4 antibodies, although such combined approaches have not been validated yet and only single-agent use is approved except of clinical trials.

### 3.2. Vaccination Strategies for Melanoma

Several strategies are currently being explored to find an effective vaccine-based therapy for melanoma, including those that have the capability to target melanoma cells directly, DC-based vaccines, peptide-based vaccines, and vector-based vaccines [49, 63].

Vaccines targeting melanoma cells are an active, specific immunotherapy based on the use of patients' own or donors' melanoma cells from resected tumors [64].

DCs are antigen-presenting cells with an elevate capacity of inducing T cell immunity through the activation of cytotoxic T cell response and proinflammatory cytokine response. DC-based vaccines have limited efficacy since tumors tend to reside in immunosuppressive microenvironments [65–67].

Viruses can infect cells and stimulate the immune response. Vaccine viruses act as oncolytic agents by activating the immune system against tumors through the production of cytokines and other immunomodulatory molecules [68]. Several oncolytic viruses have been developed based on viruses such as adenovirus, herpes simplex virus (HSV), reovirus, retrovirus, vesicular stomatitis virus, and measles virus [69]. It has been reported that replication-competent HSV in which the neurovirulence is inactivated leads to cell death in human melanoma cell lines in vitro and selectively replicates in melanoma tissue in nude mice [70]. These viruses have also been shown to be safe in phase I clinical trials by intratumoral injection in glioma and melanoma patients [70]. The main advantage of the oncolytic virus therapy is that virus replication not only directly acts on tumor cells but also disseminates the therapeutic agent further through the tumor tissue. The aim of ongoing research is to increase the tumor-selective replicative capability of the virus and its immune stimulating ability to provide a multi-modal cancer therapy.

DNA-based vaccines have been shown to be safe and immunogenic in clinical trials; however, to date, they have not shown satisfactory effectiveness [71, 72].

### 3.3. Adoptive Cell Therapy

**Figure 3:**
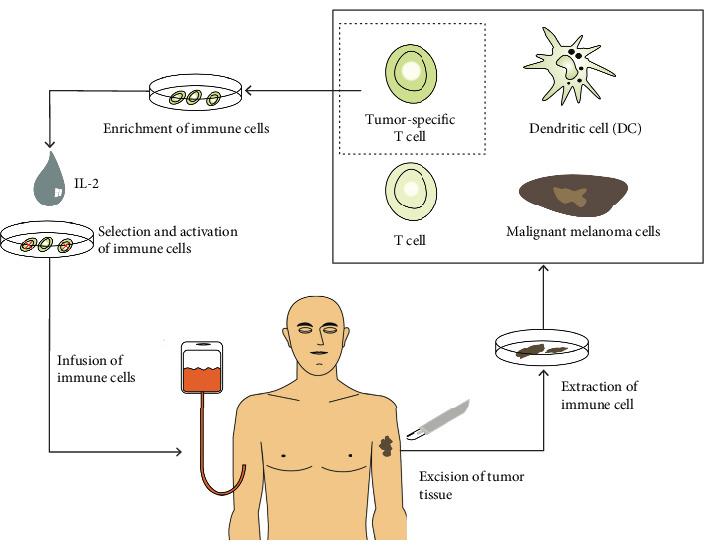
Principle of adoptive cell therapy. From Halama et al., Advanced Malignant Melanoma: Immunologic and Multimodal Therapeutic Strategies. J Oncol. 2010 [73].

Good results came from the use of adoptive cell therapy (ACT), although this is still at an experimental level and requires further validation before being considered a safe and efficacious strategy. ACT is the collection of lymphocytes from the blood or tumor of the patient and their selection, expansion, and activation in vitro. The processed lymphocytes are then infused to the patient to induce an immune anticancer response [39, 53, 73, 74]. A schematic of adoptive immunotherapy is shown in Figure 3.

The cells that are most commonly used for ACT are peripheral blood lymphocytes or TILs and LAKs [39, 74]. A novel approach of ACT is the infusion of isolated and expanded autologous CD4+ T cells previously activated using the melanoma-associated antigen (NY-ESO-1) [75]. These therapies require the development of a specific therapeutic plan with a “custom made drug” for each patient; furthermore, they require weeks of cell culture, skilled personnel, and patient preparation.

### 3.4. Immune Checkpoint Blockade

The progression of a correct immune response is characterized by some immunological checkpoints that prevent unwanted and harmful self-directed activities that lead to autoimmunity [76, 77]. Therapies developed to overcome these mechanisms by blocking the inhibitory checkpoints allow generating antitumor activity alone or in synergism with other therapies. In melanoma, these therapies target molecules that are pathologically overexpressed in melanoma such as PD-1 or CTLA-4 [78–82].

CTLA-4, which is a member of the CD28 superfamily, is induced after CD28 binding and activation. B7-1 and B7-2 are the specific ligand of CTLA-4. The interactions between CTLA-4 and activated T cells lead to another downregulator signal, blocking IL-2 transcription and so the progression through the cell cycle [4, 83–86]. The most important molecule that blocks CTLA-4 is Ipilimumab [87]; studies have shown promising results with this molecule and durability of the response, even when the treatment was discontinued [88]. Ipilimumab, a human monoclonal IgG1 antibody against CTLA-4 given at a dose of 3 mg/kg every 3 weeks for four times, represents the first FDA-approved immune checkpoint inhibitor in metastatic melanoma [87].

PD-1 is a cell-surface molecule with inhibitory properties expressed by activated T and B cells and natural killer lymphocytes that downregulates the effector function [28, 53, 89]. Studies have proven the increase of PD-1 in melanoma, which means a strong downregulation of activated T cells that helps the maintenance of tumor cells [32, 90, 91]. Nivolumab and Pembrolizumab target the interaction between PD-1 and its ligands PDL-1 and PDL-2; in melanoma, PDL-1 expression is enhanced by the presence of interferon-gamma-secreting lymphocytes from the microenvironment. Many trials have studied the efficacy of Nivolumab and Pembrolizumab in melanoma especially in comparison with Ipilimumab [92–96] and have shown a significant clinical efficacy. More recently, Gambichler et al. focused on the importance of the assessment of circulating PD-1+ regulatory T cells to predict the treatment response to PD-1 blockers such as Nivolumab and Pembrolizumab. The authors showed that circulating PD-1+ Tregs rapidly decline after the initiation of treatment with PD-1 blocking antibodies with a reduced risk for disease progression and metastatic disease [97].

Food and Drug Administration (FDA) approved Nivolumab as a single agent for patients with BRAF V600 wild-type unresectable or metastatic melanoma and in combination with Ipilimumab for patients with melanoma with lymph node involvement or metastatic disease who have undergone complete resection. Similarly, Pembrolizumab has been approved for patients with unresectable or metastatic melanoma [98–101].

One of the new frontiers of immune checkpoint inhibition is the possibility to achieve long-term survival thanks to the memory of the immune system. In fact, immunotherapy tends to turn the tumor into a chronic disease in a percentage close to 20%; in a recent meta-analysis on nearly 5,000 patients with advanced melanoma treated with Ipilimumab, the authors showed that nearly 20% of the patients were alive at 10 years [102].

Another novel issue is the evaluation of response and the identification of the endpoints. Indeed, Ipilimumab was the first drug to show improvement in OS for over 30 years, despite its impact on the ORR and the fact that progression-free survival (PFS) did not match the survival benefits achieved [4, 17, 39, 98]. This effect of OS was also shown with Nivolumab in kidney cancer [103].

This effect could depend on one side on the stimulation of the immune system and its slower activity and on the other on the immune checkpoint inhibitor treatment, in which it was firstly observed the phenomenon of pseudoprogression [104]. Pseudoprogression is characterized by an increase in the number of cells of the immune system, rather than of tumor cells, determining the appearance of the nodal progression that can be followed by the regression of the tumor; the rate of pseudoprogression is about 10-13% [105].

### 3.5. Combination Immunotherapy

Studies have revealed that the potency of cancer therapies is in the combination of drugs. Despite the success of immune checkpoint inhibitors, only a few patients have reached durable clinical responses with monotherapy. In fact, the most successful result of these medications is the possibility of using them in combination with other immune checkpoint blockers, chemotherapy, radiotherapy, or targeted molecular therapy [32, 58, 106, 107].

Rosner et al. recently described the success of combined Ipilimumab and Nivolumab and the peripheral blood clinical laboratory variables associated with the outcome in melanoma [108]. Moreover, in 2015, Postow et al. revealed an ORR of 61% in patients treated with the association of Nivolumab and Ipilimumab in comparison to patients treated only with placebo [94]. Another study compared patient with unresectable stage III or IV melanoma treated with Nivolumab alone, Ipilimumab alone, and Nivolumab plus Ipilimumab shows that median PFS was 11.5 months in the combination group, 6.9 in the Nivolumab group, and only 2.9 in the Ipilimumab group. These results show both the efficacy of comparison therapy and the good impact of Nivolumab in the treatment of metastatic melanoma [109].

Another combination therapy that had interesting outcomes is represented by the Indoleamine 2,3-dioxygenase (IDO) inhibitors and the PD-1 blockers. IDO is a cytosolic enzyme expressed in various tissues with the ability to catabolize tryptophan to kynurenine resulting in tryptophan depletion and suppression of T cell functions. The presence of IDO in melanoma has negative prognostic implications as shown in peritumoral endothelium cells or lymph nodes. Surprisingly, the combination of IDO inhibitors and anti PD-1 antibodies has failed to demonstrate an increase in OS in patients treated with combination therapy compared to single-agent treatment [110–113].

In patients with BRAF mutation, the ideal sequence of treatment or the choice of sequence of combination is still an open issue [4, 31, 98]. The results of two large ongoing studies are awaited. The SECOMBIT is a randomized comparative three-arm study, which explores combined immunotherapy (Ipilimumab plus Nivolumab) followed by targeted combination therapy (Encorafenib plus Binimetinib) or vice versa in patients with metastatic mutated melanoma with BRAF; its design includes an 8-week induction with the targeted combination therapy, followed by combination immunotherapy, and subsequently by the target combo to progression [114, 115]. The ECOG 6134 study is a randomized phase III trial comparing Ipilimumab plus Nivolumab followed by Dabrafenib plus Trametinib versus Dabrafenib plus Trametinib followed by Ipilimumab and Nivolumab in patients with advanced melanoma.

### 3.6. Future Directions

Actually, the first-line therapeutic approach for advanced melanoma consists in immunotherapy with anti-PD1 antibodies or targeted therapy with BRAF and MEK inhibitors. Evidence is accumulating on the use of new therapeutic agents for immunomodulatory treatment such as LAG3, TIM3, OX-40, CD137, IDO, and GITR. Researches concerning fully available treatment options as well as developing new drugs are ongoing [116, 117]; however, to date, the optimal first-line treatment for advanced melanoma patients is still unknown [39].

## 4. Immune-Related Adverse Events to Immunotherapy

Although immunotherapy is a targeted therapy and therefore it is better tolerated compared to common chemotherapy, it has been associated with the emergence of a new panel of dysimmune toxicities called irAEs [9, 118–120].

To date, different irAEs following immune checkpoint inhibitor therapy have been reported [121, 122]. They include dermatologic [123, 124], gastrointestinal [125], pulmonary [126], endocrine [127–129], renal [130], ophthalmologic [131], rheumatic [132], cardiovascular [133], and hematologic [134] adverse events, although they can potentially affect any tissue [122].

Dermatologic toxicities are the most common irAEs and affect up to 50% of treated patients. They include rash, pruritus, dermatitis, vitiligo and bullous dermatitis [123, 124]. Interestingly, the development of vitiligo is associated with an improved prognosis both in early and advanced disease [34]. In particular, vitiligo development in patients with stage III or IV melanoma was associated with a regression of the tumor and prolonged survival [124, 135].

Gastrointestinal irAEs include colitis, hepatitis, and pancreatitis [125]. The most common is colitis, which usually presents as diarrhea and can affect up to 40% of patients. Hepatitis may present in up to 30% of cases and presents with an increase of transaminases. Pancreatitis, which is less common, presents with increased amylase/lipase and typical clinical symptoms [136–138].

The development of thyroid disorders such as hyperthyroidism and hypothyroidism has been reported in 6%-20% of patients treated with checkpoint inhibitors [139]. The incidence of these conditions varied among patients who received single or a combination of immunotherapeutic agents [139]; studies have shown that patients treated with a combination regimen were more likely to develop thyroid alterations although their pathogenesis is still unknown [127–129].

Pulmonary irAEs include pneumonitis and sarcoidosis [140]. In case of development of pneumonitis during treatment, immunotherapy should be discontinued [126]. Sarcoidosis is a rare pulmonary toxicity in patients receiving immune checkpoint inhibitors [140, 141].

Musculoskeletal and rheumatologic adverse events occur in 2%–12% of patients and may present as inflammatory arthritis, myalgias, myositis, and polymyalgia-like syndromes [132].

Renal adverse events have been described in 2%–5% of patients and usually occur within the first 3–10 months of anti-PD1 therapy and within 2–3 months of anti-CTLA-4 therapy [130]. Renal toxicity can present with oliguria, hematuria, and peripheral edema.

Ophthalmic irAEs may present with vision alteration, optic nerve swelling, uveitis, episcleritis, and blepharitis; they are rare and have an incidence < 1% in patients receiving immunotherapy [131].

Neurologic irAEs include myasthenia gravis, peripheral neuropathy, Guillain–Barre syndrome, encephalitis, aseptic meningitis, hypophysitis, and transverse myelitis [142]. Similar to ocular adverse events, these irAEs are rare and affect less than 1% of patients [143, 144].

irAEs affecting the cardiovascular system have an incidence < 1%, usually occur within the first month of treatment and include myocarditis, arrhythmias, pericarditis, and impaired ventricular function [133, 145].

Hematologic irAEs include autoimmune hemolytic anemia, hemolytic uremic syndrome, lymphopenia, thrombocytopenia, plastic anemia, and acquired hemophilia. These adverse events are rare [134, 146].

## 5. Biomarkers

Despite the promising results, only 20-40% of melanoma patients present long-term benefits, while the remaining 80% develop primary or secondary resistance to immune-checkpoints inhibitors [8, 90, 107]. These patients are characterized by a very short PFS and OS. To date, no reliable factors have been identified to predict response to immune checkpoint inhibitors.

PD-L1 is the biomarker that has been most extensively studied thanks to its characteristic of being expressed on both tumor and inflammatory cells. However, the determination of PD-L1 has several issues, such as the extremely high dynamic marker properties, the different immunohistochemical antibody and assay in clinical practice resulting in different cut-off points, and the evidence that biopsies may not be representative of the entire tumor [82, 92, 93]. Even if still controversial, the association of high level of PD-L1 expression on tumor cells and increased response to anti-PD-1/PD-L1 treatment has been demonstrated by several studies [147].

Tumor mutational load is a promising biomarker that has been shown to correlate with better anti-PD-1 response for both Pembrolizumab and Nivolumab and combination of Nivolumab and Ipilimumab in patients affected by lung cancer but not in melanoma patients [148]. Furthermore, promising results come from the study of T cell repertoire [147], major histocompatibility complex (MHC) status [149], Interferon Y signature [150], and immune infiltrates [151].

The microbiota composition seems to influence the response and toxicity to immunotherapy. In germ-free mice model and antibiotic-treated mice, the response to CTLA-4 is reduced and the *Bifidobacterium* increases antitumor immunity and facilitates anti-PD-L1 activity [152–154]. In two different cohorts of melanoma patients treated with anti-CTLA-4, a significant association was observed between commensal microbiome composition and toxicity [155, 156].

Recently, it was demonstrated that *Faecalibaterium* is associated with better response to immune checkpoint inhibitors in melanoma patients treated with anti PD-1 [157].

New frontiers are represented on one side by the faecal transplantation as recently demonstrated in two case reports to treat colitis induced by immunotherapy [158], and on the other side by the study of metabolic profiles of microbiota and of the functional read out of host-microbiota interaction [159].

In any case, the tumor and patient immunological status, nutritional status, and the microbiome profile should be considered to better target the new immunotherapy strategy [160].

## 6. Conclusions

Metastatic melanoma is a malignancy with a poor prognosis. The introduction of immunotherapy, alone or in combination with chemotherapy, radiotherapy, or targeted molecular therapy, has significantly changed the approach to this tumor. Nivolumab, Ipilimumab, and Pembrolizumab are the drugs that are mainly used in the clinical practice; unfortunately, immunotherapy has a specific toxicity characterized by several irAEs. Future directions in the treatment of metastatic melanoma include immunotherapy with anti-PD1 antibodies or targeted therapy with BRAF and MEK inhibitors. Evidence is accumulating on the use of new therapeutic agents for immunomodulatory treatment; however, to date, the optimal first-line treatment for advanced melanoma patients is still unknown.
